# Review of Recent Efforts in Cooling Photovoltaic Panels (PVs) for Enhanced Performance and Better Impact on the Environment

**DOI:** 10.3390/nano12101664

**Published:** 2022-05-13

**Authors:** Sami Salama Hussen Hajjaj, Ahmad Abdul Kareem Ahmad Aqeel, Mohamed Thariq Hameed Sultan, Farah Syazwani Shahar, Ain Umaira Md Shah

**Affiliations:** 1 Centre for Advanced Mechatronics and Robotics (CaMaRo), Putrajaya Campus, Universiti Tenaga Nasional (UNITEN), Jalan Ikram-Uniten, Kajang 43000, Malaysia; 2 Institute of Informatics and Computing in Energy (IICE), Putrajaya Campus, Universiti Tenaga Nasional (UNITEN), Jalan Ikram-Uniten, Kajang 43000, Malaysia; eng_shurman@yahoo.com; 3 Department of Aerospace Engineering, Faculty of Engineering, Universiti Putra Malaysia, Serdang, Seri Kembangan 43400, Malaysia; farahsyaz93@yahoo.com (F.S.S.); ainumaira91@gmail.com (A.U.M.S.); 4 Laboratory of Biocomposite Technology, Institute of Tropical Forestry and Forest Products (INTROP), UPM Serdang, Seri Kembangan 43400, Malaysia; 5 Aerospace Malaysia Innovation Centre (944751-A), Prime Minister’s Department, MIGHT Partnership Hub, Jalan Impact, Cyberjaya 63000, Malaysia

**Keywords:** solar energy, photovoltaic thermal system (PV/T), cooling materials, environment, cellulose nanocrystal (CNC), nanofluids

## Abstract

The global need for energy has grown in tandem with mankind’s development and spread. This has resulted in an increase in the use of fossil energy sources, a decline in these sources and an increase in pollution, necessitating the search for renewable energy sources. One of the important ways to reduce pollution resulting from the increasing consumption of fossil energy is to enhance the sources of solar energy, of which photovoltaic cells (PV) are one of its most important tools. Therefore, it was necessary to pay attention to improving its efficiency for it to become a promising source of clean energy. PVs turn solar energy into electricity; however, the amount of electricity generated decreases as the temperature of the cells rises in response to the sun’s heat. Cooling of the optical surfaces is one of the most important elements to consider while running solar PV systems to obtain maximum efficiency. The electrical efficiency of PVs is enhanced when suitable cooling technology is used, and the rate of cell breakdown is reduced over time, extending the life of the PV panels. There are many materials used to remove unwanted heat in PV cells, and in recent years, the focus has been on integrating nanomaterials in specific proportions with traditional cooling materials such as water to improve their thermal properties. As a bio-material that is environmentally friendly, renewable, sustainable, inexpensive and has high mechanical properties, cellulose nanocrystals (CNCs) are one of the most promising materials for improving the properties of cooling materials for cooling PV cells and improving their performance.

## 1. Introduction

Fossil fuels produce more than 80% of the world’s energy. Combustion residues of these fuels negatively affect the environment by producing acid rain and causing global warming, which increases rapidly with development and increases in the world population because of the increasing demand for energy [1,2,3], so it was necessary to search for renewable energy sources [4]. Solar energy is one of the most significant renewable energy sources since it can readily be turned into thermal and electrical energy, in addition to being sustainable, available and clean energy [5].

This paper summarizes a set of research related to enhancing the efficiency of photovoltaic cells by controlling their temperature by cooling them using different ways and materials, which strengthens their position as a clean and environmentally friendly energy source.

## 2. The Review

PV panels convert solar energy into electricity. However, if the temperature of the cells rises owing to the sun’s temperature, the output of electricity falls. Therefore, different cooling techniques were used for solar cells to control their temperature, as shown in Table 1. Many of the research papers published in the literature on improving solar energy conversion and using it more efficiently have been reviewed and classified, and some of these papers are represented in Table 2. The thermal control of photovoltaic panels is emphasized in order to improve solar energy conversion to electricity through the development of cooling methods and cooling materials.

In this work, we divide reviews of previous studies according to the reasons that prompted researchers to work on developing the performance of photovoltaic panels as a promising source of energy and a suitable alternative to fossil energy. This review is divided into three areas:

2.1 Improving the generation of clean energy by cooling techniques to reduce environmental effects.
2.2 Improving solar cell’s low efficiency.
2.3 The use of cellulose nano-crystal (CNC) nanofluids as a cooling material.

It is clear from the total research reviewed in this paper that the percentage of research interested in using nanocellulose in thermal applications, which is shown in Table 3, is low, indicating that more attention should be paid to the use of this bio-sustainable, friendly environmental material.

### 2.1. Improving the Generation of Clean Energy by Cooling Techniques to Reduce Environmental Effects

Because of the increasing demand for energy and the excessive use of traditional energy sources, this has led to an increase in environmental pollution due to emissions from burning fuels. Cooling solar cells increases their potential to create clean energy and use it as an alternative to traditional polluting energy sources.

Researchers provided an in-depth analysis of the design components of a concentrated photovoltaic thermal, heat transfer medium and new application sectors in this paper. The findings show that CPVT systems are a promising system for producing high amounts of clean electrical and thermal energy that are in line with the seven sustainable development goals by using this energy in a variety of thermal applications such as space heating and cooling, desalination, electrical energy generation, greenhouses and so on [71,72,73]. Other researchers compared the performance of a water-based photovoltaic system (thermal), a PV/T system with PCM, an air-based PV/T system and a conventional PV panel in different studies. 

In comparison to alternative kinds of cooling, it was found that the efficiency of the systems in producing energy depends on the type of material used, and all the arrangements proved to be more important solutions for delivering superior thermal and electrical efficiency systems (compared with the conventional system), thus serving as a promising source as an alternative to fossil energy that gives rise to air pollution and an increased earth temperature [48,74,75]. Some researchers focused on the increasing consumption of fossil energy and the accompanying emissions and pollution as a result of urban transformation and expansion of the construction and service sectors in developing countries in particular. Accordingly, the researchers’ efforts focused on improving the performance of photovoltaic cells, whose efficiency is affected by atmospheric conditions, to make them a suitable substitute for the production of clean energy [76,77,78,79,80].

Researchers [3,81,82] looked at the energy-increasing and environmental impacts of using nanofluids (NFs) in PVTS by measuring their physical and thermal properties. The researchers discovered that dispersion of nanoparticles in the base fluid increases the PVTS’ thermal and electrical performance, which improves the systems’ environmental characteristics. In fact, a nano-fluid-based solar system may avoid the release of greenhouse gases emissions, particularly carbon dioxide (CO_2_), into the environment more effectively than pure heat pumps by producing more energy. The performance of the integration of the Kalina cycle with CPVT for a multi-generation and hydrogen production system was investigated utilizing air and water as a cooling medium and three distinct mass flow rates. According to the findings of these studies, electricity, hydrogen and hot air production were increased. These kinds of systems would be used to minimize pollution in the environment because the emissions will decrease significantly [83,84].

The steady increase in population numbers and the need to address the problem of food insecurity in some countries made some researchers search for quick, effective and environmentally friendly ways to dry food, as the drying process consumes energy intensively, and the use of fossil energy in the drying process increases pollution. In these cases, the focus was on improving the performance of solar energy systems to provide the appropriate energy [85,86,87]. On the other hand, water is also a paramount necessity for human life, and the need to provide for drinking water in water-poor countries requires desalination since desalination consumes a large amount of energy. Some researchers have developed ways to use solar energy when desalinating water as well as storing energy for use when needed. This reduces the consumption of fossil energy and the emission of gases [88,89].

### 2.2. Improving Solar Cells’ Low Efficiency

**Figure 1:**
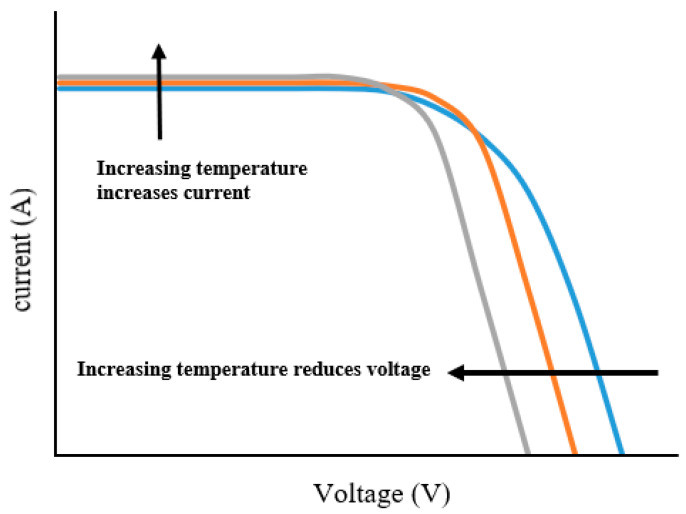
Effect of temperature on the I–V characteristic.

More than 50% of the solar radiation falling on PV cells turns into thermal energy rather than electricity [90]. This may cause a harmful consequence, such as a significant reduction in the efficiency of PV cells by approximately 0.45% per °C [34,91,92], and structural damage to the unit if heat stress persists for a long time [93]. In order to obtain high efficiency for PV cells, it is necessary to maintain a low temperature as much as possible, especially when using solar concentrators, which increase the temperature of cells very much [94]. When the temperature of solar cells rises, the maximum power output decreases (some photovoltaic cells, such as amorphous silicon and thin film cells, are exempt from this requirement); see Figure 1 [95].

Several ways for cooling the PV module, such as the PV/T air-heating manifold and water-cooled PV/T, have been introduced and used. The heat transfer process from PV modules should be improved; the operating temperature must be reduced to improve the system’s efficiency. A hybrid PV/T solar system is one method for cooling the PV panels. It consists of a cooling system connected to a solar PV panel, so the hybrid model can be considered as two different mechanisms, one providing electrical energy and the other producing heat (thermal energy), which is used to warm the cooling medium [96]. It was found that cooling PV panels with water increases the solar cell’s output power by approximately 50% approximately and keeps the surface temperature of the cell at an acceptable level [97].

There is a lot of research that has studied the possibility of cooling photovoltaic cells using different materials such as phase change materials (PCM), salts, oils, nanomaterials and metallic materials with different thermal conductivities. In the same line, many researchers have also focused on improving the performance of PV cells by cooling them via traditional methods using air and water. 

Because air is a less efficient coolant medium than water, water cooling functions at higher temperatures. It allows for more efficient waste heat recovery by utilizing it for household water heating or air conditioning. A coolant is a liquid or gas that is used to lower and regulate the system’s temperature. A good coolant has a high thermal capacity and a low viscosity that is inexpensive, chemically inert, non-toxic and does not corrode the cooling system. In order to benefit from the thermal energy absorbed from the PV panels, they are cooled and heat is extracted from them, which is reflected on their overall efficiency and increases their electrical efficiency, which is negatively affected by heat.

Researchers worked to address the effect of the efficiency of photovoltaic cells on temperature by working on cooling them in different ways. Cooling by air was one of the first and simplest methods used, which a group of researchers used in different ways and conditions. The results indicated a modest perspective because thermal air capacity is low, but there are advantages to the use of air, as it is used directly in buildings without heat exchange; the problems of fluid leakage are also not as critical when using air, and the problems of corrosion and freezing do not exist [48,98,99]. Air’s thermal properties make it less efficient as a coolant medium, so water cooling operates at higher temperature levels and allows waste heat recovery to be more efficient because of its high thermal capacity, which encouraged a large number of researchers to use water in innovative and multiple ways to cool photovoltaic cells. As a consequence, the final findings imply that installing water-cooled PV panels is an appealing and realistic alternative for improving short- and long-term efficiency, with a significant influence on the PVs’ power growth and durability [41,46,100,101,102]. The development of using traditional materials (air and water) as cooling materials in different ways and forms, and the possibility of obtaining different amounts of energy according to the amount of coolant flow, led to an expansion in the use of the resulting thermal energy according to the need for thermal applications [31,44].

As seen in Table 4 from the following research, the researchers add different nanomaterials to a base fluid in different quantities to determine the best concentration of nanoparticles for their application. The results showed that nanofluids have higher conductivity when the fluid density and viscosity increase slightly compared to the base fluid, and the higher the nanomaterials’ percentage in the base liquid, the more thermal the conductivity becomes.

So, examination of the performance of photovoltaic cells cooled with nanofluid improves the heat transfer coefficient, PV power and PV efficiency and therefore improves the overall efficiency of the system [34,42,47,90,113]. Other researchers have used the base and nanofluids materials as spectral beam splitting cells by installing a layer of coolant on the surface of the photovoltaic panel so that it cools it by absorbing excess heat and filters solar radiation by allowing only visible radiation to reach the photovoltaic cell and convert to electricity. They noticed that nanofluids were a good filter and water also has the best qualities, and it is easily available to compare with glass filters; in addition, the results demonstrated a considerable increase in the amount of energy generated by this suggested system as well as an increase in overall efficiency [114,115,116].

Some researchers successfully developed tools to increase the performance of solar cells by using concentrated solar energy to obtain the largest amount of solar radiation that can be received from these cells. The increase in solar radiation is accompanied by an increase in temperature, and thus cooling the cells is very important in these cases to avoid damage and a sharp decline in their efficiency. Multi-junction solar cells are also chosen for high concentration ratios due to their good performance at high operating temperatures. The results showed that high sun radiation and the volume flow rate of the cooling fluid have a significant influence on PVT performance, according to the research [43,117,118,119].

### 2.3. The Use of Cellulose Nano-Crystal (CNC) Nanofluids as Cooling Materials

Nanofluids are the most recent advancement in modern technology for enhancing the performance of engineering equipment and machinery [120]. The nanomaterial is dispersed with the basal fluid to prepare the nanofluids [121]. In general, chemical and physical synthesis techniques are used to produce nanoparticles [122]. Unfortunately, scientists have noted that the use of nanomaterials poses severe issues regarding toxicity and safety, particularly because the most-well-studied quantum dots (QDs) include hazardous components including CdSe, CdTe and CdS [123]. So, nano cellulose has received increased attention in recent years as a result of some of its significant qualities, including its biodegradability, excellent mechanical capabilities, decreased density, abundance, and most importantly, an eco-friendly aspect [124]. Nano cellulose can be considered an excellent material for the development of high-performance nanocomposites.

CNCs have attracted the attention of business and academia due to their unique qualities, which include their cheap cost, renewable resource extraction, minimal toxicity and high mechanical properties. This made some researchers conduct studies to find out the possibility of improving the thermophysical properties of cooling fluids by adding crystal nano cellulose at various concentrations and temperatures to them and using these materials in different applications. The results experimentally and theoretically indicate that thermal conductivity increases as the CNC volume concentration and temperature increase, so CNC is a promising material in industrial, electronic, and thermal applications [60,63,64]. Some researchers used experimental and one-dimensional modelling software to forecast the performance characteristics and efficacy of cellulose nanoparticles integrated with Ethylene Glycol as a coolant with varying percentages and compared it to pure water. The results revealed that using nano cellulose with Ethylene Glycol for automobile radiator applications had a higher thermal absorption effectiveness than using distilled water and that the heat transfer coefficient of cellulose nanofluids increased as the radiator size grew, so employing cellulose nanoparticles in conjunction with ethylene glycol as a coolant in automotive radiators is acceptable and enhances the heat transfer efficiency [61,62].

Furthermore, nano cellulose and aluminum oxide hybrid nanofluids as a novel coolant for car radiators was examined experimentally by Naiman et al. (2019). The thermal conductivity of the resultant fluid improved significantly, and the thermal conductivity of Al_2_O_3_/CNC composite nanofluids enhancement improved with the temperature and volume concentration, according to the researchers [35].

Other researchers have been interested in incorporating nanocellulose with other compounds to form nanocomposites and have studied their properties and investigated their performance as a superior heat transfer nanofluid than base fluid coolant at various concentrations and temperatures as depicted in Table 5. The incorporation of nano cellulose has generally improved nanocomposites’ thermal and thermomechanical properties. The hybrid nanofluids may be used to replace standard heat transfer fluids, resulting in more efficient thermal structures. They also have good aqueous solubility in the base liquid. [65,66,67]. Farhana et al. (2021) studied the addition of nanoparticles (Al_2_O_3_ and CNC) to the basic fluid of a solar collector and compared their effects on its thermal performance. They came to the conclusion that flat plate solar collectors’ energy gains and thermal efficiency had increased. For (0.5 percent) Al_2_O_3_, the greatest efficiency was approximately (2.48 percent), while for (0.5 percent) CNC nanofluids, it was around (8.46%) [58].

## 3. Summary and Evaluation

**Figure 2:**
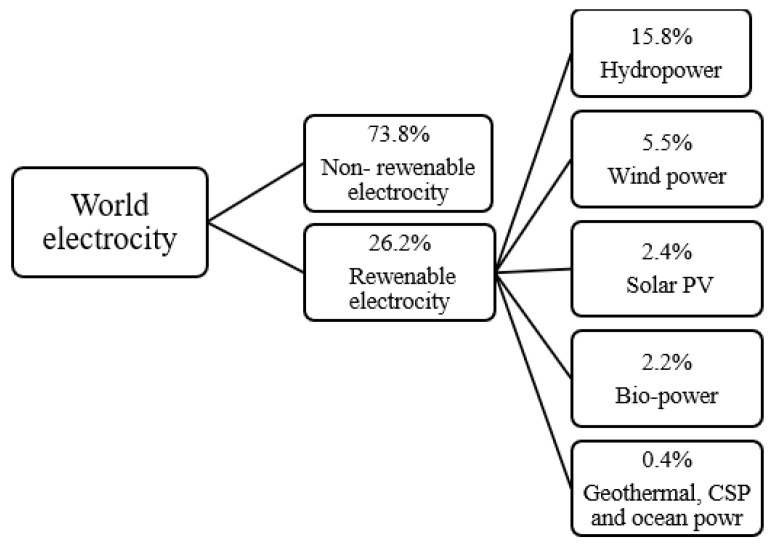
Renewable energy shares of global electricity production.

From the previous studies, it is clear that the use of PV cells has become one of the best ways to produce clean electric power in recent years and to increase the production of electricity while reducing the cost of solar systems and reducing the area on which the PV panels are installed. Even though interest in renewable energy sources has grown in recent decades, solar energy’s proportion of worldwide energy (power) output remains below 2.5 percent [125], as shown in Figure 2.

The temperature of the PV cell rises when the radiation intensity is high, lowering its efficiency and increasing the risk of damage. Therefore, it is necessary to cool these cells by exposing them to a coolant medium (coolant system).

After a thorough examination of the subject of PV cells and their evolution over the previous decades, as well as previous studies that looked into different ways to improve the efficiency of PV systems and different methods and materials to cool them so that their temperature does not exceed the required limit, water is one of the best materials used for cooling because of its availability, easy storage, cheapness and the fact that it can be used in relatively high pressures and temperatures and has a high thermal capacity.

However, it is possible to work on improving water’s mechanical and thermal properties by adding nanomaterials to it, which will positively affect its performance. This also applies to other cooling materials so that their properties can be improved by adding certain materials to them or merging them with each other. 

There are many tools and materials used to get rid of unwanted heat in PV cells, and in recent years, the focus has been on integrating nanomaterials in certain proportions with traditional cooling materials such as water to improve their thermal properties. The use of nanomaterials in cooling depends on their availability, price and environmental friendliness. Its efficiency by improving the properties of cooling liquids depends on the size of its components, the volume ratio and its stability in the liquid, in addition to the temperature.

Cellulose nanocrystal (CNCs) are one of the promising materials in improving the properties of cooling materials that can be used in PV cells’ cooling, and this is reflected in the performance of it, as this material is considered a bio-material that is environmentally friendly, renewable, sustainable, inexpensive and has high mechanical properties.

## Figures and Tables

**Table 1:** Photovoltaic cooling techniques.

Techniques	Advantages	Limitations
Air cooling Photovoltaic/Thermal [6,7,8,9,10]	Easy-to-use technology. Air is always accessible. Improves the overall efficiency. It is economically feasible. Heated air is employed in HVAC systems. Reduces corrosive danger.	Has limited thermal capacities and requires a lot of energy to circulate air blowers (in active cooling). Has low mass-flow rates, so little effect on PV temperatures.
Water cooling Photovoltaic/Thermal [11,12,13,14]	Overall efficiency has improved. Increased electric energy conversion efficiency. Hot water is utilized for residential purposes. Space requirements are less than for individual systems.	High start-up costs. System life is reduced. In chilly weather, it is possible that you will freeze. Pumping power consumes a lot of electricity. Possible corrosion, fouling and leaking.
PV/water spraying [15,16,17,18]	Increased conversion of solar energy. Higher thermal conductivity and heat capacity (low thermal resistance).	The PV panel’s surface area is partly cooled. A higher price (maintenance, pumping power) Heat is a waste of resources.
PV/water immersion cooling [19,20,21]	Extremely effective. Friendly to the environment Both the front and rear surfaces transmit heat.	The depth of submersion has an impact on efficiency. Higher price. Because the item is insulated inside the water, the system is complicated to build.
PV/Phase-Change Materials cooling [10,22,23]	At modest temperature changes, huge amounts of heat may be stored. Phase-change happens at a steady temperature; therefore, the system can work even when the sun is not shining. The heat that is absorbed can be utilized to heat structures.	PCM has a low heat conductivity in its solid form. Some PCMs are poisonous and provide a fire hazard. After the conclusion of the life cycle, there is a difficulty with disposal. The quantity of active volume available for thermal storage is limited by segregation.
Cooling of PV/Heat Pipes [24,25,26,27]	Heat fluxes that are extremely high. Heat exchange that is passive. Transfer of heat across large distances. It is simple to combine. Longer life span.	High price. Difficult to produce. Non-condensable gas production. Working agent leakage.
PV/Microchannel heat sink cooling [28,29,30,31,32]	Removes a lot of heat from a tiny space. Low inventory of fluids is necessary. Low electricity consumption; thermal resistance is low.	Limitations on pressure decrease. Corrosion is an issue. Manufacturing at a high cost.
PV/Nano-fluids cooling [33,34,35,36,37]	There are nanofluids on the market. Thermal efficiency that is higher.	Technology in its infancy. Influences are unknown (interaction with base fluids and characteristics). Nanoparticles are expensive.
PV/Spectrum filter [38,39,40]	The operating temperature has been reduced. Hybridization with concentrating or other systems is possible.	Technology that is not completely developed. High-priced (glass filters)

**Table 2:** Cooling technique methods.

Cooling Technique	Method/Paradigm	Finding/Results
Forced water [41]	Comparison of the overall efficiency between covered and uncovered PVT.	The overall annual efficiency of covered PVT is 42.3% and 52.6% for uncovered one.
PV/T Nanofluids [42]	Numerical and experimental in the current work, a comprehensive assessment of nanoparticle applications in photovoltaic (PV) cooling was conducted.	Photovoltaic systems can benefit from nanofluids to improve their thermal, electrical and overall efficiency.
CPV/T cooling mediums [43]	A thorough literature analysis is offered, outlining the benefits, drawbacks and promise of current CPVT research. There are graphical and tabular summaries of several distinct CPVT design offers in this section.	Multijunction, non-silicon-based solar cells are chosen for high concentration ratios due to their good performance at high operating temperatures. Silicon-based monocrystalline solar cells, on the other hand, are favored at lower concentration ratios due to their cost-effectiveness and off-the-shelf availability.
CPV/T cooling mediums [44]	Previous studies show the temperature of the cooling medium for photovoltaic cells (low, medium, high) and the applications in which this heat can be used. In addition, it shows the type and efficiency of the photovoltaic cells used and the type of working fluid.	PVT analysis using inexpensive and readily accessible spectrum filters liquids (UV–VIS–NIR). Electrical and thermal efficiency averages 12.53 percent and 47 percent, respectively.
PV/T spectrum filter [40]	A UV–VIS–NIR spectrophotometer is used to analyze the absorption and transmission of 200–2500 nm wavelength solar spectrums of various liquids	The output power, conversion efficiency and total energy efficiency of the proposed combination system are higher than those of a standard solar concentration PV system under the same conditions, as per the outcomes
Forced air [45]	Using a varied flow rate of air to cool PV cells and comparing its efficiency to conventional PV	Thermal efficiency improved from 30.3 percent to 46 percent, while electrical efficiency increased from 9.5 percent to 10.2 percent, according to the findings. In comparison to the traditional cell, the flow increased from 0.021 kg/s to 0.042 kg/s
Forced water [46]	Experimental examination of the performance of a photovoltaic panel under the influence of different solar rays with stabilization of the amount of coolant flow and the inlet temperature of the coolant.	High sun radiation and the volume flow rate of the cooling fluid have significant influence on PVT performance, according to the research.
PV/T Nanofluids [47]	investigate the achievement of a nanofluid-based PV/T collector using various nanofluids, particle volume concentrations and mass flow rates.	1: The PV/T collector with Al_2_O_3_/water as a coolant has a greater electrical and thermal power than one with TiO_2_/water. 2: Nanoparticle concentration improves the nanofluid heat transfer coefficient, PV power and PV efficiency.
Forced air [48]	Experimental performance of the PVT (air) and mathematical models were compared with commercial PV.	The results showed an improvement in the performance of air-cooled photovoltaic cells, with great agreement between the theoretical and experimental results
PV/heatsink [49]	The thermal efficiency sinks for various designs with varying fin thicknesses and fin heights, as well as their effect on PV, were evaluated using a simulation model.	For CPV cells, a multi-channel heat sink is used. The conversion efficiency was around 32 percent, and the net power was around 4050 W, thanks to a 91 °C cell temperature and 0.6 m/s flow velocity.
PV/heat pipe [50]	The thermal performance of two systems (wickless heat pipe PV/T and wire-meshed heat pipe PV/T) operating at varying inclination degrees was investigated experimentally using a solar simulator.	At no reduction in the temperature, wickless and wire-meshed heat pipe PV/T systems’ thermal efficiencies were 52.8 percent and 51.5 percent, respectively.
PV/heatsink [51]	Different degrees of the heatsink’s fin width and their influence on hydraulic performance, as well as the temperature level and uniformity of the PV module, are investigated using numerical simulations using CFD software.	The suggested heatsink design has superior hydraulic properties, resulting in increased heat transmission while utilizing the same amount of material as a traditional design.
PV/heat pipe [52]	During the day and at night, the heat loss coefficients of Formal PV/T and PV with heat pipe systems are inversely proportional. Based on data from a typical climatic year in Shanghai, the impacts of each on the annually successful supply days, yearly heat gain and yearly electric gain are compared and analyzed in this study.	The PV/heat pipe system’s decreased night-time heat loss greatly enhances photo thermal performance while somewhat lowering photovoltaic performance when compared to a normal PV/T system.
PV/spraying water [53]	When compared to a steady-spray water cooling system and an uncooled PV module, a pulsed-spray water cooling system is developed for solar panels to enhance efficiency and reduce water usage throughout the cooling process.	The results demonstrate that the solar panel’s highest electrical energy generation improves by roughly 33.3 percent, 27.7% and 25.9%, respectively, as compared to non-cooled panels while using spray water cooling (steady and pulsed) and non-cooled panels.
PV/spraying water [54]	To better understand the heat transfer characteristics between the solar panel and water, the impact of the water spray’s mass percent on heat transfer coefficients was investigated by calculating the best quantity of total spray water to use for cleaning and cooling PV.	At an optimal flow of 170 L/h, the electrical efficiency of the system was 15.73 percent, with a panel energy capacity of 40.25 W, and the system produced a peak power of 39.48 W, while the pump needed 0.77 W of power
PV/immersion [55]	For linear concentrating photovoltaic systems, cooling by the immersion of solar cells in dimethyl silicon oil is suggested as a heat diffusion solution.	The electrical behavior of the silicon-oil-immersed cells is steady, and after 270 days, no significant efficiency deterioration was found.
PV/immersion [20]	A dish concentrator (250X) with tracking was used to test a unique CPV system that used de-ionized water for cooling by immersing.	The temperature distribution of the module is very uniform; however, after a wide time of immersion in de-ionized water, the cell module’s electrical efficiency drops.
PV/PCM [56]	To lower the working temperature, solar panels using phase-change materials (PCM) are employed.	PV–PCM integrated systems have been shown to increase electricity efficiency by up to 5%. According to the findings, inorganic PCMs offer a high potential for PV cooling.
PV/PCM [57]	Under hot climatic circumstances, this study illustrates how to use paraffin wax (PCM) to reduce the running temperature of a c-Si PV module.	The use of a PCM improves the performance of a c-Si PV panel by controlling its thermal properties, which is critical for its reliable operation in hot regions.

**Table 3:** Nanocellulose in thermal applications.

Application	Challenge/Question	Materials Used	Finding/Results
Improve the solar collector’s efficiency [58]	Low energy efficiency and low output temperature in solar collectors.	Addition of nanoparticles (Al_2_O_3_ and CNC) to a basic fluid.	The flat plate solar collector’s energy gain and thermal efficiency were both increased. For 0.5 percent Al_2_O_3_, the greatest efficiency was 2.48 percent, while for 0.5 percent CNC nanofluids, it was 8.46 percent. Using CNC/water-EG nanofluid in a flat plate solar collector, an increase in efficiency of around 5.8% may be attained.
Strengthening the thermo-physical characteristics of the solar collector’s fluid [59]	Base fluids are suffering from low thermal physical properties.	Using nano cellulose at different concentrations and different temperatures.	Because the nanofluid displays greater thermal conductivity and viscosity at elevated temperatures, it may be deduced that it is suitable for use in a higher-temperature environment.
Improve machining performances [60]	Flank wear, chipping and abrasion are the most common causes of MWF machining failure.	Adding cellulose nanocrystal at different concentrations and different temperatures to cooling fluid.	As the CNC volume concentration and temperature grow, the thermal conductivity of the base fluid increases, enhancing its influence on the cutting machine.
In a heat exchanger, nanocellulose is used as a heat transfer liquid [61]	Investigate the performance of car radiator coolant using a cooling material consisting of cellulose nanoparticles combined with Ethylene Glycol.	As a coolant, cellulose nanoparticles combined with Ethylene Glycol were used.	It may be observed that employing cellulose nanoparticles in a car radiator as a cooler in combination with ethylene glycol is practical and improves the heat transfer rate.
Impregnate nano-cellulose with EG for car radiator applications [62]	Nanoparticles improve the convective heat transfer performance of the base fluid.	As a coolant, cellulose nanoparticles combined with Ethylene Glycol were used.	When compared to distilled water, the use of nanocellulose with Ethylene Glycol for automobile radiator applications demonstrates a higher heat absorption effectiveness.
Using cellulose nanocrystals in different thermal applications [63]	Increase the use of crystal nanocellulose in a variety of applications.	Adding crystal nanocellulose to certain materials.	CNCs have attracted the attention of business and academia due to their unique qualities, which include cheap cost, renewable resource extraction, minimal toxicity and good mechanical strength.
Thermal applications [64]	In most technical applications, the low heat transfer capability of conventional thermal transport fluids is an unresolved problem.	Addition of crystal nanocellulose materials to the base liquid.	Thermal conductivity and relative viscosity increase with the volume concentration of nanoparticals and the temperature.
Coolant of automotive engine radiator [35]	Enhance radiator performance.	Enhance the thermo-physical properties of base fluid by adding nanomateials to it with different volume concentrations and different temperatures.	The thermal conductivity Al_2_O_3_/CNC composite nanofluids’ enhancement increases with the temperature and volume concentration.
Car radiator application [65]	The rapid rise in energy demand needs additional improvements in the heat transfer process as well as a decrease in energy loss owing to inefficient system operation.	Changing percentages of hybrid metal oxides just like Al_2_O_3_ and TiO_2_ both with and without CNC taken from the plant base.	The mono Al_2_O_3_ nanofluids outperformed the CNC and TiO_2_ nanofluids in terms of thermal conductivity enhancement. The Al_2_O_3_/CNC nanofluids, on the other hand, had better thermal conductivity than Al_2_O_3_/TiO_2_ nanofluids (mono and hybrid)
General thermal application [66]	Because of its exceptional mechanical characteristics, renewability and biodegradability, nanocellulose has gotten a lot of interest in research and industry in recent years as a nanoscale material for reinforcing polymer matrix composites.	Incorporating nanocellulose with other compounds to form nanocomposites.	Nanocellulose has enhanced the thermal and thermomechanical properties of nanocomposites in general.
Radiator for automobiles [67]	In numerous engineering processes, standard working fluids (such as water, motor lubricant and ethylene glycol) have a limited ability to transmit heat.	Graphene nanoplatelets (GNPs) and cellulose nanocrystal (CNC) spread in a base fluid.	The colloidal stability of GNPs/CNC nanofluids at a 0.1 percent volume concentration was exceptional in the base fluid of EG: W at a 60:40 ratio. Traditional heat transfer fluids may be replaced with the present hybrid nanofluid, resulting in more efficient and compact thermal structures.
Thermal applications [68]	Invent nanofluid with the highest thermal conductivity and specific heat capacity.	CNC (nanomaterial) distributed in a combination of ethylene glycol and distilled water.	The density has a proportionate relationship to volume concentration and an inverse relationship with temperature, according to the results of the experiments. The specific heat capacity, on the other hand, has a proportionate relationship with temperature and an inverse connection with volume concentration.
Machining process cooling [69]	To increase product quality and the cutting equipment’s life, it is critical to reduce heat generated during machining.	CNC is dispersed in a combination of ethylene glycol distilled water.	When CNC-based nanofluids are utilized, the total heat generated at the cutting tool and the temperature created at the chip when milling both improve dramatically.
Machining process cooling [70]	Coolant handling and disposal costs are considerable, and with the potential of harmful materials, the disposal of spent coolant is a major issue, since it may have a negative impact on the environment.	Nanofluid made of ethylene glycol and nanocellulose.	The greatest temperature measurement achieved with MWF is 225 °C, but the greatest temperature reading produced with nano-fluid is 154 °C, reflecting a decreased temperature distribution for the chip developed during milling.

**Table 4:** Using nanomaterials in thermal applications.

Materials Used	Problem/Challenge	Concentration	Finding
Add nano-carbon to KAl(SO_4_)_2_·12H_2_O/Na_2_SO_4_·10H_2_O	Energy shortage	1 wt% nano-carbon	The proposed combination is low-priced, easy-to-prepare and accessible, lowering the cost of an energy storage device [103].
Dissolve KAl(SO_4_)_2_·12H_2_O in distilled water	Improving the optical specifications of the water.	0.05%, 0.075%, 0.1%, 0.125 and 0.15 gm./mL	The optical characteristics of these solutions are shown to vary continuously with an increasing concentration in this investigation. Because the ultrasonic absorption coefficient rises for the reason of concentration, it may be employed as a coating for moving bodies to detect using ultrasonic technology [104,105].
Coupled KAl(SO_4_)_2_·12H_2_O with GN	Improving the thermal conductivity of the KAl(SO_4_)_2_·12H_2_O.	0.5%, 1%, 1.5%, 2%, 2.5%	The conductivity of KAl(SO_4_)_2_·12H_2_O/GN compounds increases considerably as the GN concentration increased [106].
Use nano-(Al_2_O_3_, CuO and SiC) with water	When the temperature of solar cells rises, their efficiency falls.	0.5, 1, 2, 3 and 4%	The results revealed that when nanoparticles were introduced to water, thermal conductivity rose, and SiC nanofluid had a higher stability than the other nanofluids investigated [36].
Al_2_O_3_-water nanofluid	Enhancing the PV/T overall efficiency of the system.	0.05, 0.075, 0.1, 0.2, 0.3	The average amount of electrical energy of nanofluid-based PVTS rises by 16.3, 24.6 and 17.1 percent for 0.05, 0.1 and 0.3 percent, respectively [107].
Al_2_O_3_, TiO_2_ and ZnO distributed in water	Enhancement of energy, exergy and entropy generation compared to the PV unit.	0.2 wt.%	PVT/ZnO and PVT/TiO_2_ systems have greater total energy and exergy efficiency than other systems [108].
Cellulose nanoparticles Ethylene glycol Water	Low energy efficiency and low output temperature plague solar collectors.	0–1.3%	Because the nanofluid displays greater thermal conductivity and viscosity at elevated temperatures, it may be deduced that it is suitable for use in a higher temperature environment [58].
Zn-water	During the summer season, heat is retained inside the PV cells.	0 to 0.5% wt.	When compared to water-based PVTS, the electrical energy of Zn–water nanofluid-based PVTS increased by 20% [109].
MWCNT/water	PVT’s thermal performance should be improved.	0 to 1% wt.	PVTS using MWCNT and water had about 0.14 and 3.67 percent greater TE and EE than pure water. If you compare it to fresh water, the findings of the experimental and numerical tests indicated that using MWCNT/water increases the overall power efficiency by 4.11 and 3.81 percent, respectively [110]
GNP/water	PV electric generation decreases when the temperature of photovoltaic (PV) panels continues to rise.	0 to 0.15% volume fraction	The NF volume proportion and the HTF flow rate were varied between 0 and 0.15 percent and 20–40 LPM, respectively. According to their findings, the PVTS’s overall efficiency improved by 14.1, 12.6 and 10.9 percent at flow rates of 40, 30 and 20 LPM, respectively [111]
Boehmite water	For the PV cell, evaluate the cooling performance using water-based Boehmite nanofluids.	0.01, 0.1 and 0.5 wt.%	When nanoparticles are present, the average temperature of the PV cell is significantly lower than that of the base fluid [112].

**Table 5:** Nanocellulose in combination with other substances.

Added Nanomaterial Type	Properties	Finding
Graphene nanoplatelets and CNC [67]	GNPs with characteristics of an 800 m^2^/g specific surface area, 99.9% purity, 3 mm size and 1.5 µm diameter were employed. Volume concentrations (0.1% to 0.2%)	For 30 days, the examined nanofluids remained stable, with no significant sedimentation. The findings of GNPs/CNC nanofluids at a 0.1 percent volume concentration showed outstanding colloidal stability in the base fluid of EG:W at a 60:40 ratio.
Al_2_O_3_, TiO_2_ and CNC [65]	The sizes of the smaller and larger particles are 50–90 nm and 1–5 µm, respectively. Volume concentrations (0.1%, 0.5% and 0.9%) Temperatures ranging from 30 °C to 70 °C	The mono Al_2_O_3_ nanofluids outperformed the CNC and TiO_2_ nanofluids in terms of thermal conductivity enhancement. The Al_2_O_3_/CNC hybrid nanofluids, on the other hand, had better thermal conductivity than the other mono and hybrid nanofluids (Al_2_O_3_/TiO_2_).
Al_2_O_3_/CNC composite nanofluids are made in a 60:40 ratio [35]	Volume concentrations are 0.1%, 0.5% and 0.9%. Temperatures ranging from 30 °C to 70 °C	With an increasing temp. and volume fraction, the thermal conductivity of Al_2_O_3_/CNC composite nanofluids improves.
Al_2_O_3_/CNC distributed in a base mixture water to ethylene glycol (40:60) [61]	Nanoparticles’ average size is 13 nm with a spherical shape. CNC volume concentrations are 0.1%, 0.5%, 0.9% and 1.3%	The rate of heat transfer rises as the flow rate of the coolant or working fluid in the radiator cooling system increases.
Al_2_O_3_ and CNC [58]	Using 0.5% Al_2_O_3_ and 0.5% CNC, volume fractions are 0.1%, 0.3% and 0.5%. CNC crystal diameter from 9 to 14 nm	The thermal conductivity of nanofluids rose, but viscosity decreased as the temperature climbed. Nanofluids have the potential to improve the efficiency of flat-plate solar collectors.
Nano cellulose, ethylene glycol and water [59]	It was carried out at temperatures of 30–70 °C and volume concentrations of up to 1.3 percent.	It can be concluded that the nanofluid is applicable in a higher temperature environment since the nanofluid exhibits enhanced thermal conductivity and viscosity at the elevated temperature.

## Data Availability

Not applicable.
